# Killing Two Birds With One Stone: Successful Management With Rituximab in the Coexistence of Myasthenia Gravis and Marginal Zone Lymphoma

**DOI:** 10.7759/cureus.45877

**Published:** 2023-09-24

**Authors:** Xiao Li, Carlos Gaibor, Mark Fesler

**Affiliations:** 1 Internal Medicine, St. Luke's Hospital, Chesterfield, USA; 2 Hematology and Oncology, St. Luke's Hospital, Chesterfield, USA

**Keywords:** paraneoplastic syndrome, immunotherapy, rituximab, marginal zone lymphoma, myasthenia gravis

## Abstract

Myasthenia gravis (MG) is an autoimmune neuromuscular disorder characterized by motor weakness affecting various muscle groups. The simultaneous or sequential occurrence of lymphoma and MG, often seen in patients, could be influenced by common genetic and biological factors that drive unregulated lymphocyte growth, leading to autoimmunity and lymphoma. This case report describes a 72-year-old male with coexisting marginal zone lymphoma and MG, who exhibited a positive response to rituximab treatment intended for the lymphoma, but surprisingly effective for both conditions. The patient experienced significant improvement in MG symptoms and a decline in serum anti-acetylcholine receptor (AChR) antibodies, along with achieving hematologic remission in the lymphoma. These observations emphasize the potential therapeutic advantages of rituximab in treating cases with concurrent MG and lymphoma, providing useful insights for future research and multifaceted clinical management.

## Introduction

Myasthenia gravis (MG) is an autoimmune neuromuscular disorder characterized by fluctuating motor weakness involving ocular, bulbar, limb, or respiratory muscles [1]. It results from an immunologic attack on proteins in the postsynaptic membrane of the neuromuscular junction, primarily targeting acetylcholine receptors (AChR) and receptor-associated proteins. Approximately 80 to 90% of MG patients have detectable autoantibodies against AChR in their serum [1]. A subset of MG patients who test negative for AChR antibodies may harbor antibodies against other muscle membrane constituents, such as muscle-specific receptor tyrosine kinase (MuSK) or intracellular muscle proteins like the ryanodine receptor, titin, myosin, and alpha-actin [2,3].

Anticholinesterase agents and intravenous immune globulin (IVIG) offer rapid, short-term symptom control in MG, but most patients eventually need chronic immunotherapy for sustained disease stability. While glucocorticoids are typically the first-line immunosuppressive treatment due to their rapid effect, nonsteroidal immunotherapy is crucial for patients who show insufficient response to glucocorticoids, cannot reduce their glucocorticoid dosage without symptom recurrence, or experience toxicity from long-term glucocorticoid use [4].

While thymomas are commonly associated with MG and necessitate evaluation in all patients, MG has also been linked to extrathymic tumors, such as small-cell lung cancer and Hodgkin lymphoma [5-7]. However, the precise nature of this correlation remains undetermined. Meanwhile, the coexistence of MG and tumors will make the treatment more complex.

In this study, we present a noteworthy case of MG occurring in a patient diagnosed with marginal zone lymphoma, who exhibited a favorable response to rituximab treatment intended for the lymphoma, but was surprisingly effective for both conditions.

## Case presentation

**Figure 1:**
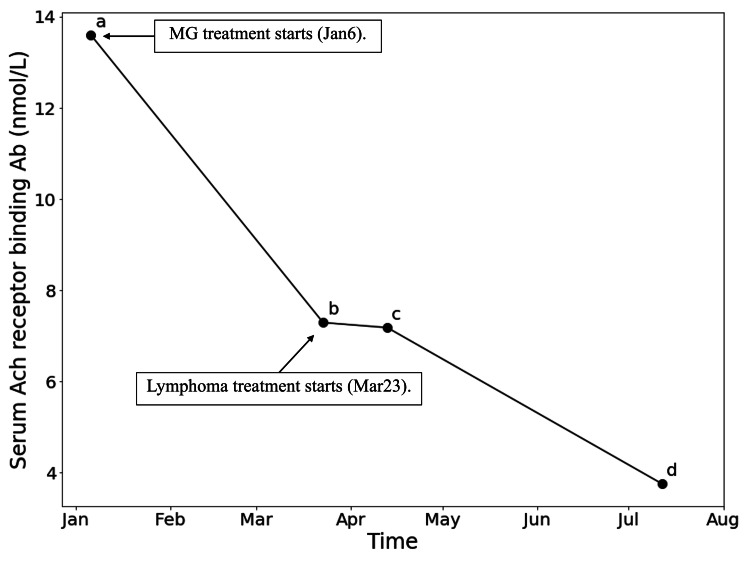
Serum levels of AchR antibodies decreased after MG and lymphoma treatment. a). MG treatment with pyridostigmine and prednisone, b). First dose of rituximab, c). Forth dose of rituximab, d). First maintenance dose of rituximab. AchR: acetylcholine receptor, MG: myasthenia gravis

A 72-year-old male with a history of marginal zone lymphoma diagnosed in 2014 was asymptomatic and had not received any treatment under his primary care physician (PCP) follow-up. In January 2023, this patient presented with a three-week history of intermittent right eye ptosis, slurred speech, and facial weakness. Physical examination revealed significant right eye ptosis, worse than the left eye, along with moderate dysarthria and bifacial weakness on direct confrontation. Lab tests revealed positive AChR antibodies with a binding antibody titer of 13.6 nmol/L (Figure 1), and MuSK antibody testing yielded negative results. MG was confirmed and CT chest ruled out thymoma. The patient was treated with pyridostigmine 60 mg three times daily and prednisone, which was titrated from 20 mg daily to 40 mg daily. Subsequently, the patient experienced significant symptom improvement, with only mild residual right eye ptosis at the end of the day.

Patients with MG typically need to shift to steroid-sparing agents like azathioprine and mycophenolate mofetil (MMF) for long-term treatment, which carries an elevated risk of malignancies. Given this patient’s coexistence of MG and lymphoma, an oncology consultation was sought for further evaluation. Laboratory revealed an elevated white blood cell count (WBC) of 105.2 K/uL and a hemoglobin level of 14.2 g/dL (Table 1). Abdomen and pelvis CT scans were unremarkable, while peripheral blood smear analysis showed absolute lymphocytosis, primarily consisting of small mature-appearing lymphocytes with round to slightly irregular nuclei and scant cytoplasm. Flow cytometry and genetic analysis confirmed the diagnosis of marginal zone lymphoma.

**Table 1:** Clinical course: CBC levels with differential changes following rituximab treatment. CBC: complete blood count, WBC: white blood cells

CBC with differential	2023-03-23	2023-03-30	2023-04-07	2023-04-13	2023-07-12	Reference range
WBC (K/uL)	105.2	9	8.9	10.5	11.7	4.3 - 10.0
Hemoglobin (g/dL)	14.2	14.9	13.6	14.4	14.1	13.6 - 16.5
Platelet (K/uL)	201	169	157	178	180	140 - 350
Neutrophil (K/uL)	11.6	5.0	5.7	5.8	6.4	1.9 - 7.0
Lymphocyte (K/uL)	91.5	3.1	2.0	3.6	4.3	0.7 - 4.5
Basophil (K/uL)	0.0	0.0	0.0	0.1	0.0	0.0 - 0.2
Eosinophil (K/uL)	0.0	0.1	0.3	0.1	0.1	0.0 - 0.7
Monocyte (K/uL)	2.1	0.6	0.8	0.8	0.8	0.1 - 1.3
Neutrophil (%)	11	56	64	55	55	40 - 60
Lymphocyte (%)	87	34	23	34	37	20 - 40
Monocyte (%)	2	7	9	8	7	2 - 8
Eosinophil (%)	0	1	3	1	1	1 - 4
Basophil (%)	0	1	1	1	0	0.5 - 1

Considering the coexistence of MG and lymphoma, the patient was recommended to receive rituximab at 375 mg/m2 weekly for four weeks for lymphoma treatment. Remarkably, following the first dose of rituximab, his WBC dropped from 105.2 K/uL to 9.0 K/uL (Table 1), indicating a positive therapeutic response. After the four-week rituximab treatment, the patient achieved normalized absolute lymphocyte count and kappa/lambda ratio (Table 2), indicative of hematologic remission. The patient was advised to undergo rituximab maintenance every three months as part of the post-treatment plan.

**Table 2:** Kappa/Lambda ratio normalized after rituximab treatment.

Lab results	2023-01-24	2023-07-12	Reference range
Kappa light chain, free	30.8	18.5	3.3 - 19.4
Lambda light chain, free	12.0	13.9	5.7 - 26.3
Kappa/Lambda ratio, free	2.57	1.33	0.26 - 1.65

During the treatment course, the patient's MG symptoms were closely monitored. Prior to rituximab administration, the patient was maintained on acetylcholinesterase and prednisone at a daily dose of 40 mg, which was tapered down to 20 mg daily during the rituximab treatment period. Notably, following the rituximab infusion, the patient experienced a marked improvement in his MG symptoms, with no MG complaints reported after the second rituximab dose. Additionally, a decline in serum anti-ACh antibodies from 7.29 nmol/L to 3.75 nmol/L was observed (Figure 1), further supporting the positive impact of rituximab on MG.

## Discussion

MG is a B-cell-mediated autoimmune neuromuscular disorder characterized by skeletal muscle weakness and fatigability. Immunotherapy plays a crucial role in MG treatment, with glucocorticoids being the initial choice. For generalized MG patients, nonsteroidal immunotherapeutic agents are often added for maintenance to reduce long-term glucocorticoid toxicity. Among these agents, azathioprine and mycophenolate mofetil (MMF) are commonly used glucocorticoid-sparing therapies for AChR antibody-positive or seronegative MG [4].

However, the therapeutic benefits of azathioprine and MMF need to be carefully weighed against their potential risks. Notably, the prolonged use of these agents has been associated with an elevated risk of developing malignancies, including hematologic cancers such as lymphomas [8-10]. In the context of our patient, who already has a history of marginal zone lymphoma, the use of azathioprine and MMF was deemed inappropriate due to their oncogenic potential.

The synchronous or asynchronous co-occurrence of lymphoma and MG, as seen in many cases, is thought to be driven by shared pathways and genes that foster unchecked lymphocyte proliferation, possibly resulting in both autoimmunity and lymphoma. There is also evidence suggesting that MG may function as a paraneoplastic syndrome of lymphoma [6]. Despite the absence of typical indications for lymphoma treatment in this patient, treatment was recommended by the oncologist to improve his autoimmune condition and to avoid steroid toxicity.

Rituximab, a monoclonal antibody that targets the CD20 antigen on B-lymphocytes, is often the preferred systemic therapy for most symptomatic marginal zone lymphoma cases. While azathioprine and MMF are the typical treatment options for MG patients, studies also show rituximab’s efficacy in treating MG, particularly in MuSK-positive disease [4]. In this case, rituximab was chosen for its intended effect on the lymphoma and was found to improve the patient's MG symptoms as well. The positive response to rituximab observed in this case led to the remission of both the lymphoma and MG symptoms.

## Conclusions

This case report presents a compelling instance of coexisting MG and marginal zone lymphoma successfully managed with rituximab. The treatment not only achieved hematologic remission in lymphoma but also led to significant improvement in MG symptoms and a reduction in anti-AChR antibodies. These findings highlight the potential therapeutic advantages of rituximab in cases of concurrent MG and lymphoma, suggesting a promising avenue for future research and treatment approaches in such complex clinical scenarios.
